# Optimization of Sweet Potato (*Ipomoea batatas* L.) Chlorogenic Acid Extraction Process and Hypoglycemic Effect Study

**DOI:** 10.3390/plants15010120

**Published:** 2026-01-01

**Authors:** Xiaofei Wang, Jiayu Zhang, Chen Yang, Xiaohan Yu, Dan Tian, Mingli Han, Na Xiao

**Affiliations:** 1 College of Agronomy, Shandong Agriculture University, Tai’an 271018, China; 19276195647@163.com (X.W.); 13165389975@163.com (J.Z.);; 2 National Key Laboratory for Tropical Crop Breeding & Haikou Key Laboratory for Research and Utilization of Tropical Natural Products, Institute of Tropical Bioscience and Biotechnology, CATAS, Haikou 571101, China

**Keywords:** sweet potato (*Ipomoea batatas* L.), chlorogenic acid crude extract, network pharmacology, molecular docking, response surface testing, hypoglycemic effect

## Abstract

Chlorogenic acid (CGA) is an active ingredient widely found in plants and has been shown to have potential blood-glucose-lowering effects. However, the research on the efficient extraction processes of CGA from sweet potatoes and its systematic mechanisms underlying hypoglycemic effects is still insufficient. This study optimized the extraction of CGA from various sweet potato parts and varieties using ethanol, and predicted the hypoglycemic mechanism of sweet potato leaves via network pharmacology and molecular docking. The efficacy of the leaf extracts was demonstrated through the in vitro inhibition of hepatic glucose output, accompanied by minimal cytotoxicity, and was further validated in an acute mouse model. The results demonstrated that the optimal extraction conditions were an ethanol concentration of 65.48%, a liquid–solid ratio of 39.00 mL·g^−1^, an ultrasonic time of 50.00 min, and a temperature of 45 °C. The final extraction yield of CGA crude extract was 3.54%, with the highest content in sweet potato leaves, suggesting a multi-target synergistic mechanism of action for sweet potato leaves. Further in vitro experiments indicated that the CGA crude extract can exert hypoglycemic effects by inhibiting hepatic gluconeogenesis. In conclusion, the study lays a foundation for the further purification and utilization of sweet potato CGA, and establishes a theoretical basis for the development of sweet potato leaf resources as hypoglycemic functional ingredients.

## 1. Introduction

Sweet potato (*Ipomoea batatas* L.) is the world’s seventh-largest crop. According to statistics from the Food and Agriculture Organization (FAO) [1], China cultivated sweet potatoes on a total area of 2,750,400 hm^2^ in 2022, with a total fresh weight production of 5,945,500 tons. The total cultivation area and production accounted for 30% and 56% of the world’s total, respectively. As the world’s leading producer of sweet potatoes, China conserves more than 2000 conserved sweet potato germplasm resources [2]. Research by Wang Zhen et al. [3] demonstrated that sweet potato stems and leaves contain high levels of chlorogenic acid (CGA), with the content in leaves harvested in September reaching as high as 4.49%. Despite their high nutritional and functional value and the capacity for multiple annual harvests, a substantial proportion (95–98%) of sweet potato stems and leaves is discarded as waste, with only a minor fraction (2–5%) utilized as animal feed, leading to substantial resource waste [4]. According to the literature, there are more studies on polyphenols [5,6] and substances such as anthocyanins in sweet potato stems and leaves and fewer studies on CGA. In view of the waste of resources where 95–98% of sweet potato stems and leaves are discarded, based on existing research, advanced methods such as response surface methodology (RSM) are used to systematically optimize the CGA extraction process, aiming to achieve breakthroughs in higher extraction efficiency and lower cost.

Diabetes mellitus, a severe global public health threat and the third leading cause of death among chronic non-communicable diseases, significantly impairs human health [7]. A key pathophysiological hallmark of type 2 diabetes is elevated hepatic glucose output, which raises fasting and postprandial blood glucose levels, largely attributable to insulin resistance [8]. However, clinical management has mainly focused on the insulin pathway, while the role of glucagon was ignored, a hormone promoting hepatic glucose production [9,10]. In this context, natural products from sweet potato have shown promising bioactive potential for managing metabolic disorders. According to the reported studies, phenolic acids, cellulose, anthocyanins, polysaccharides, carotenoids, vitamins, proteins, and flavonoids extracted from sweet potatoes demonstrated biological activities in tests of bioactive components in studies worldwide [11,12,13]. Beyond their established antioxidant and anti-inflammatory properties [14,15], sweet potato constituents demonstrate specific hypoglycemic activity. For instance, sweet potato polysaccharides can improve insulin resistance and induce islet regeneration [16,17]. Notably, polyphenols from sweet potato upregulate the PI3K/AKT/GSK3 signaling pathway in the liver and the PI3K/AKT/GLUT4 pathway in muscle, thereby enhancing the activity of enzymes related to glycogen synthesis and glucose metabolism [18,19]. These findings align with the paradigm of network pharmacology, which utilizes systems biology to elucidate multi-target mechanisms of natural products [20], suggesting that sweet potato extracts likely exert effects via synergistic, multi-target mechanisms rather than a single pathway.

CGA, a dietary phenolic acid, exhibits its diverse biological activities, including anti-inflammatory, antihypertensive, antitumor, antioxidant, and glucose metabolism-regulating effects [21,22,23]. While most studies on CGA’s hypoglycemic mechanism focus on the insulin signaling pathway, its role in modulating the glucagon pathway remains underexplored. CGA has been shown to inhibit glucose-6-phosphatase, the key enzyme catalyzing the final step of gluconeogenesis, and inhibits hepatic glucagon response to reduce fasting blood glucose in mice [24,25,26]. CGA extracts from *Cecropia obtusifolia* and *Cecropia peltata* can improve hyperglycemia by specifically blocking hepatic glucose output and regulating glucose homeostasis through pathways such as PI3K-AKT [27,28]. Building on this potential and the aforementioned research, this study employed an integrated approach combining network pharmacology, molecular docking, and experimental validation, to examine how CGA from sweet potato leaves exerts its hypoglycemic effect by inhibiting glucagon-regulated hepatic glucose production.

Our study aimed to optimize the extraction of CGA from sweet potato stems and leaves using response surface methodology. The extraction yield of the CGA crude extract was the evaluation index, with ethanol concentration, liquid–solid ratio, and ultrasonic time as independent variables. The objectives were to establish an efficient extraction protocol and evaluate the hypoglycemic potential of the crude extract, thereby promoting the comprehensive utilization of sweet potato resources and reducing waste, as well as to provide new ideas for the prevention and treatment of diabetes by CGA.

## 2. Materials and Methods

### 2.1. Materials and Animals

The sweet potato plant material was sourced from Tai’an, Shandong Province. The six newly grown leaves at the top of the stem and vine are young, and the six leaves at the bottom are old. The older leaves, younger leaves, and stems were dried, crushed, sieved (100 mesh), sealed in a brown paper bag, and placed in the refrigerator.

SPF-grade 18–22 g male C57BL/6J mice, C60-sterilized laboratory animal feed, C60-sterilized laboratory animal bedding were utilized in this study. All operations were carried out in strict accordance with the relevant regulations on laboratory animal ethics. All experiments and animal care were approved by the Animal Ethics Committee of Shandong Agricultural University (SDAUA-2023-025). The mice were housed in an animal facility with constant temperature (22 ± 1 °C) and humidity, following a 12 h light/dark cycle, with free access to water and food, and regular changes.

For the pyruvate tolerance test, 24 fasted mice were randomly divided into four groups (blank, pyruvate, CGA crude extract, met) with six mice in each group, and fasting blood glucose was measured. The blank and pyruvate groups were given 10 mL·kg^−1^ of normal saline, the other groups to be tested were given 150 mg·kg^−1^ of CGA crude extract or 200 mg·kg^−1^ of metformin (Met). After 0.5 h, the blank group was injected with normal saline, and the other three groups were intraperitoneally injected with 2 g·kg^−1^ pyruvate, and a change in blood glucose within 2 h was observed. The area under the curve (AUC) for blood glucose was calculated as 0.5 × (glucose_0 h_ + glucose_0.5 h_)/2 + 0.5 × (glucose_0.5 h_ + glucose_1 h_)/2 + (glucose_1 h_ + glucose_2 h_)/2. The selection of CGA crude extract treatment dosage and safety evaluation are based on previous research conducted by our research team [26].

### 2.2. Extraction Process of Chlorogenic Acid from Old Leaves of Sweet Potato

Based on previous literature on the extraction of CGA from dandelion [29], an ultrasonic-assisted method was selected for CGA extraction. Precisely 0.50 g of leaf powder was weighed into a 25 mL ground-glass conical flask. A specified volume of absolute ethanol was added, and the mixture was subjected to ultrasonication in an ultrasonic bath (SB-5200DTD, Ningbo Xinzhi Biotechnology Co., Ltd., Ningbo, China) at 45 °C, 90 W, and 40 kHz for a predetermined duration. The water bath temperature was maintained by circulating water and monitored with a thermometer. The flask was secured to the rack in the water bath using a rubber band. Following centrifugation at 5000× *g* for 15 min, the supernatant was collected as the CGA crude extract.

### 2.3. Single-Factor Experimental Design

The single-factor experiment was performed by following the method of Mangiapelo et al. [30] to investigate CGA extraction yield under different extraction conditions. The extraction yield was employed as the metric to evaluate the CGA crude extract from sweet potato leaves using a single-factor experimental design, as outlined in Table 1.

### 2.4. Response Surface Experimental Design

Based on the previous experiments, a three-factor and three-level experimental design was carried out according to the Box–Behnken design principle, and the specific process is shown in Table 2 to determine the optimal process conditions for the CGA crude extract in sweet potato. Each group performs ten repetitions.

### 2.5. Chlorogenic Acid Standard Curve Plotting

The absorbance of CGA standard solutions at various concentrations (0, 0.002, 0.004, 0.006, 0.008, 0.010, 0.012, 0.014, 0.016 g·L^−1^) was measured at a wavelength of 331 nm [31]. A standard curve was then established using the least squares method, yielding the linear regression equation A = 70.796C1 − 0.0068, R^2^ = 0.9992. A is the absorbance, and C1 is the CGA concentration, g·L^−1^.

### 2.6. Calculation of Chlorogenic Acid Extraction Rate

Using anhydrous ethanol as a blank control, the absorbance of CGA crude extracts was measured by colorimetry, and the content of CGA crude extracts was calculated based on the standard curve. The extraction yield of CGA was calculated using the following formula: yield of CGA/% = C2 × V × n/m. C2: Calculated concentration of CGA standard, mg·mL^−1^; V: ethanol volume, mL; n: dilution factor; m: weight of sweet potato powder, mg.

### 2.7. Determination of CGA Crude Extracts of Sweet Potato Leaf by HPLC

Method changes were made based on previous research [32]. Use Dionex Ultimate 3000 high-performance liquid chromatography (HPLC) system (Thermo Fisher Scientific, Waltham, MA, USA) to establish a gradient elution system. Chromatographic conditions: chromatographic column: COSMOSIL 5C18-MS-II (4.6 × 250 mm, Nacalai Tesque, Kyoto, Japan); mobile phase: 10% acetonitrile-water (0.1% phosphoric acid), filtered through a 0.45 μm microporous membrane and degassed by ultrasound before use; column temperature: 35 °C; flow rate: 1 mL/min; injection volume 10 μL. CGA standards of 20, 40, 60, 80, 100, and 1000 μg/mL were prepared, and the standard curve was plotted with the peak area (mAU*s) as the vertical coordinate and the concentration (μg·mL^−1^) as the horizontal coordinate, and then the CGA crude extract was analyzed by the same method in HPLC, and its concentrations were calculated.

### 2.8. Collection of Active Components and Targets of Sweet Potato Leaves

Chemical components of sweet potatoes were collected through the Traditional Chinese Medicine Systems Pharmacology Database and Analysis Platform (TCMSP). Screening was conducted based on an oral bioavailability (OB) threshold ≥30% or a drug-likeness (DL) threshold ≥0.18. The TCMSP platform was also used to identify the target proteins of the active components of honeysuckle.

### 2.9. Screening of Disease Targets for Diabetes

Target genes associated with diabetes were retrieved from the human gene compendium (GeneCards, https://www.genecards.org/, accessed on 19 November 2025) and the Online Mendelian Inheritance in Man (OMIM, https://www.omim.org/, accessed on 19 November 2025) databases. The retrieved targets were integrated, and duplicate entries were removed. The targets related to the active ingredients were then mapped against the disease targets, and a Venn diagram was plotted to identify the potential therapeutic targets for diabetes.

### 2.10. Construction of Protein-Protein Interaction (PPI) Network and Screening of Core Targets

Using the data obtained in Section 2.9, apply Cytoscape 3.10.3 to construct a “drug-active ingredient-target gene-disease” relationship network to analyze the mechanism of sweet potato leaves in treating diabetes. Import the obtained intersecting targets into the STRING platform (https://cn.string-db.org/, accessed on 19 November 2025), set the species to “Homo sapiens” and the confidence level to 0.400, construct a PPI network, and save the resulting data.

### 2.11. Gene Ontology (GO) and Kyoto Encyclopedia of Genes and Genomes (KEGG) Pathway Enrichment Analysis

The selected potential targets were subjected to functional enrichment analysis, including Gene Ontology (GO) and Kyoto Encyclopedia of Genes and Genomes (KEGG) pathway analyses, via the DAVID database. The results of these analyses were subsequently visualized as bubble plots.

### 2.12. Component–Target Molecular Docking

The compound’s 3D structure (PubChem) and AKT1, EGFR, STAT3, TNF, GAPDH structure (PDB: 2OCB, 3P0V, 6TLC, 1RJ7, 1ZNQ, RCSB) were prepared in AutoDockTools-1.5.7 by removing solvents/ligands and adding hydrogens. Protein–ligand docking conformations were rendered using PyMOL (https://pymol.org, accessed on 19 November 2025).

### 2.13. Isolation of Primary Mouse Hepatocytes

Primary mouse hepatocytes were isolated by an improved two-step collagenase perfusion, as previously described [33]. Briefly, fasted C57BL/6J mice were anesthetized with 1% sodium pentobarbital. Livers were then perfused and digested using collagenase type IV solutions (Liquid I and II). Isolated hepatocytes were filtered, centrifuged, and cultured in DMEM with 10% FBS on 48- or 6-well plates.

### 2.14. Cytotoxicity Assay

Primary hepatocytes were plated at a density of 1 × 10^5^ cells per well in a 24-well plate; after attachment, medium was replaced with serum-free DMEM for starvation. Cells were cultured overnight, and 5 replicate wells were incubated with different concentration CGA crude extract for 24 h. MTT solution was added, culture was continued, supernatant was removed, dimethyl sulfoxide was added, and the absorbance value was measured at 490 nm by shaking at 37 °C at low speed for 10 min.

### 2.15. Glucose Output

Primary hepatocytes were plated at a certain density (60–70%) and cultured overnight. Prior to the assay, cells were serum-starved in KRH solution for 2–4 h. The cells were then incubated for 6 h with the relevant substrates (10 mM pyruvate, 100 nM glucagon, or 1, 10, 100, 1000, 10,000 μg/mL CGA crude extract) in KRH solution, and the absorbance was measured using a glucose assay kit (Shanghai Rongsheng Biopharmaceutical Co., Ltd., Shanghai, China) at 505 nm.

### 2.16. Data Statistics and Analysis

All experiments were repeated at least three times, the results were analyzed by IBM SPSS Statistics software (version 25.0; IBM Corp., Armonk, NY, USA) and expressed as mean ± standard deviation, the statistical differences between the groups were evaluated by one-way ANOVA, the differences were considered to be statistically significant at *p* < 0.05, and the statistical data were plotted using OriginPro 2021 and GraphPad Prism 8 software. The extraction optimization was analyzed using Design-Expert 13 software.

## 3. Results and Discussion

### 3.1. Single-Factor Test Results

**Figure 1:**
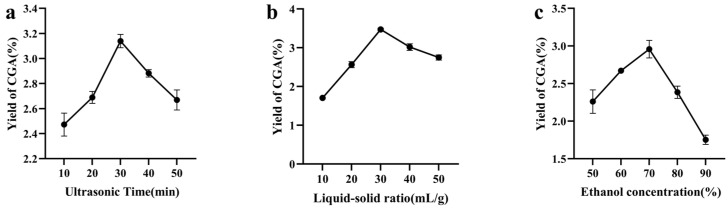
The effect of single factor on the extraction yield of chlorogenic acid from sweet potato. (**a**): ultrasonic time; (**b**): liquid-solid ratio; (**c**): ethanol concentration.

The effects of ultrasonic time, liquid–solid ratio, and ethanol concentration on the extraction yield of chlorogenic acid in sweet potato were investigated. As can be seen from Figure 1a, the CGA yield peaked at 30 min. Shorter times (e.g., 10 min) were likely insufficient for complete dissolution, while prolonged ultrasonication (e.g., 50 min) may have promoted the extraction of impurities or potential degradation of CGA [34]. Consequently, 10, 30, and 50 min were chosen as the levels for the subsequent experimental design. As can be seen from Figure 1b, the extraction yield of CGA was highest under the condition of a liquid–solid ratio of 30 mL·g^−1^, and then it showed a decreasing trend, which may increase the surface area of the ethanol solution and the diffusion rate of CGA. Therefore, the liquid–solid ratios of 10, 30, and 50 mL·g^−1^ were selected for the response surface test. As can be seen from Figure 1c, when the ethanol volume fraction was 70%, the extraction of CGA reached its peak, and at low ethanol concentrations, some of the CGA was not dissolved. Under the high concentration of ethanol, it precipitated other compounds or induced undesirable interactions with CGA. Therefore, 50%, 70%, and 90% ethanol concentrations were selected for the response surface test.

### 3.2. Response Surface Test Results

#### 3.2.1. Significance Test and Variance Analysis of Regression Model

In the response surface test, we obtained a quadratic multiple regression equation as follows (Table 3):

Y = −2.8524 + 0.0060A + 0.0215B + 0.1791C + 0.0010AB + 0.0003AC − 0.0001BC − 0.0009A^2^ − 0.0006B^2^ − 0.0014C^2^

Based on the results in Table 4, the results indicate that the *p*-value of the model was less than 0.01, indicating that the regression equation was highly significant. The lack of fit was 0.0863, which was not significant, indicating that there was no obvious lack-of-fit factor in the equation. This indicates that the fit of the experimental model was good, thus validating the feasibility of the experimental approach. According to the results of the *F* value, it could be seen that the extraction yield of CGA in older leaves of sweet potato was affected by the linear terms C > B > A, among which the linear item C had the greatest effect on the extraction yield of CGA extract crude, and the linear item A had the smallest effect. In addition, AB, C, A^2^, and C^2^ had an extremely significant effect on the extraction rate, *p* < 0.01; the quadratic term B^2^ had a significant effect on the extraction yield of CGA, *p* < 0.05; and the linear terms A and B and the interactions AC and BC had no significant effect (*p* > 0.05).

#### 3.2.2. Responsive Surface Experimental Design

**Figure 2:**
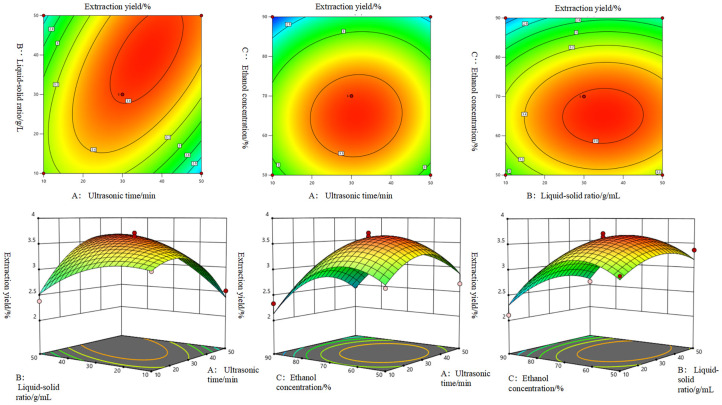
Response surface result.

In the 3D surface response plots (Figure 2), the contour lines form an elliptical shape with a close distribution, and the color gradient intensifies from light green to dark red. These features indicate a strong interaction between the corresponding two factors. Furthermore, the steep slope of the three-dimensional surface plot suggests that these factors have a significant influence on the extraction yield of the CGA crude extract.

The optimal extraction conditions were as follows: ethanol concentration of 65.48%, liquid–solid ratio of 39.00 mL·g^−1^, ultrasonic time of 50.00 min, and setting temperature of 45 °C, under which the extraction yield of CGA crude extract was 3.54%. Based on the optimal conditions, three replicate validation experiments were performed, as shown in Table 5. The extraction yield of the crude chlorogenic acid obtained was 3.51–3.56%, with no significant difference from the predicted value.

#### 3.2.3. Determination of CGA Crude Extract in Sweet Potato Leaves

**Figure 3:**
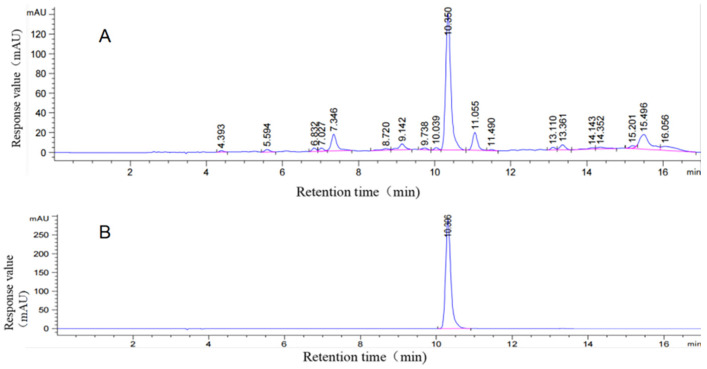
Content of crude extract of chlorogenic acid in sweet potato leaves. (**A**) HPLC of crude extract of chlorogenic acid from sweet potato leaves; (**B**) HPLC of chlorogenic acid standard.

The CGA crude extract content in sweet potato leaves was quantified by HPLC, and the results are shown in Figure 3. Using the regression equation from the standard curve (y = 2.8155x − 16.8910), the concentration was calculated to be 479.48 μg·mL^−1^. This high CGA content confirmed the presence of the target compound in the extract and validated its suitability for subsequent experiments.

### 3.3. Comparison of CGA Crude Extracts in Different Parts and Varieties

**Figure 4:**
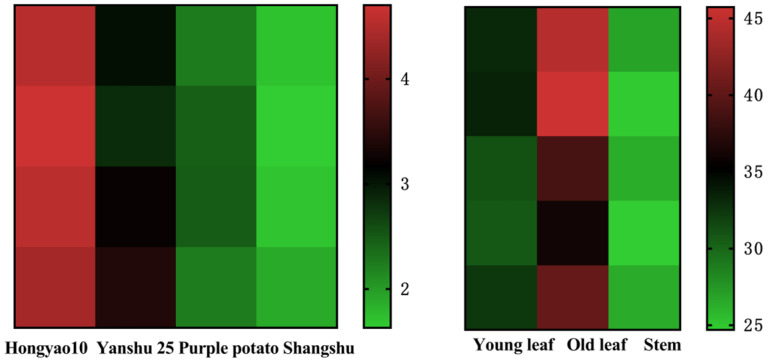
Chlorogenic acid in different parts (younger leaves, older leaves, and stems) and varieties (Hongyao 10, Yanshu 25, purple sweet potato, and Shangshu) of sweet potato.

CGA was extracted from three different parts of sweet potato (younger leaves, older leaves, and stems) and four different varieties (Hongyao 10, Yanshu 25, purple sweet potato, and Shangshu) (Figure 4), and it was found that the CGA crude extract in sweet potato was the highest in the old leaves, and the CGA crude extract in Hongyao 10 was the largest. Sweet potato leaves are rich in CGA, which requires further processing and comprehensive evaluation as a by-product.

### 3.4. Analysis of Network Pharmacology Results

#### 3.4.1. Screening of Active Components, Targets of Sweet Potato Leaves, and Disease Targets

**Figure 5:**
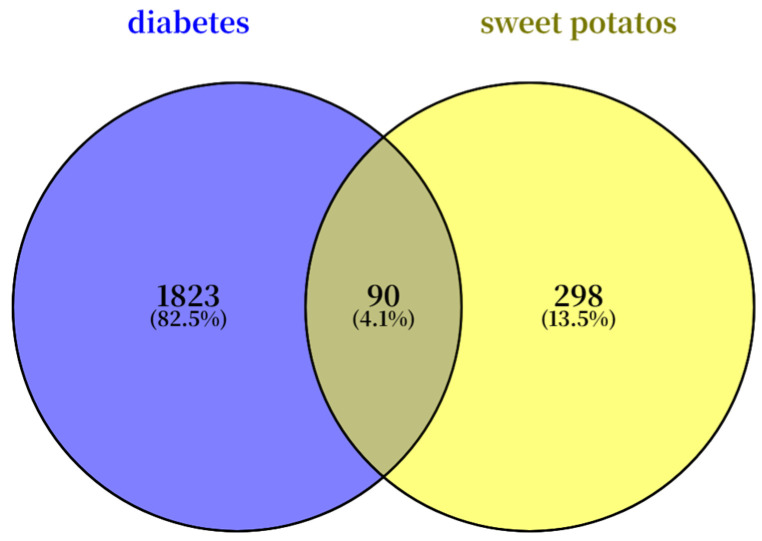
Venn diagram of overlapping genes between sweet potato leaves and diabetes.

The common chemical components of sweet potato leaves were screened through the TCMSP platform based on OB ≥ 30% or DL ≥ 0.18, resulting in nine chemical components. The first nine chemical components are shown in Table 6. From the disease target database (GeneCards and OMIM databases), 1823 disease-related targets were identified, while 298 target predictions were associated with the active compounds based on the drug target database. Among these, 90 overlapping targets were identified as potential therapeutic targets for the treatment (Figure 5).

#### 3.4.2. Results of the “Drug-Active Component-Target-Disease” Network Analysis

**Figure 6:**
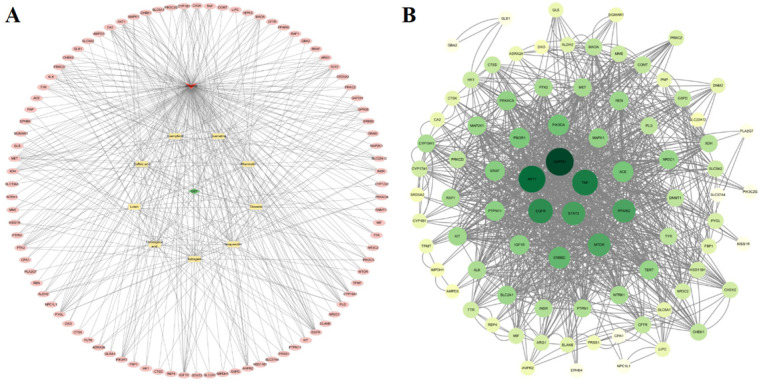
Sweet potato leaves–active ingredients–target genes–diabetes network diagram (**A**) Squares: active compounds of the sweet potato leaf; circles: targets; diamonds: the sweet potato leaf; arrows: diseases; visualization of the interaction network between sweet potato leaves and diabetes-related proteins (**B**).

Using these overlapping targets from Section 3.4.1, a PPI network comprising 101 nodes and 429 edges was constructed (Figure 6A). Multiple active compounds of the sweet potato leaf can act on multiple different targets, reflecting its characteristic of multi-component, multi-target synergistic therapy. Visualize the core targets in the PPI network according to their degree values (Figure 6B). The larger the degree value, the higher the corresponding node, and the darker color indicates that the target protein is more important. Core targets GAPDH (108), AKT1 (100), TNF (92), EGFR (84), and STAT3 (74) are prominent nodes in the network and may be potential targets for sweet potato leaf treatment of diabetes.

#### 3.4.3. Gene Ontology (GO) and Kyoto Encyclopedia of Genes and Genomes (KEGG) Functional Enrichment Analysis

**Figure 7:**
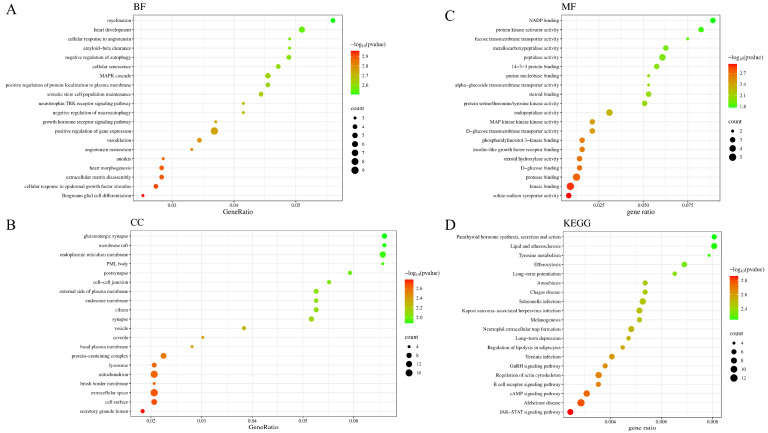
Results of GO (**A**–**C**) and KEGG (**D**) functional enrichment analysis.

A total of 156 GO terms were significantly enriched with a significance level of *p* < 0.05. It mainly included 135 biological processes (BP, Figure 7A) such as myelination, negative regulation of autophagy, MAPK cascade, and positive regulation of gene expression. The cellular component (CC, Figure 7B) process was mainly related to functions such as the synapse, endoplasmic reticulum membrane, and vesicle. It mainly included molecular functions (MFs, Figure 7C) such as NADP binding, protein kinase activator activity, *α*-glucoside transmembrane transporter activity, and steroid binding. Sweet potato leaves may exert their effects by regulating these biological processes. KEGG signaling pathways arranged by *p*-value are shown in Figure 7D. Lipid and atherosclerosis, tyrosine metabolism, cAMP signaling pathway, JAK-STAT, and other signaling pathways were associated with diabetes. These results indicated that sweet potato leaves treat diabetes through the synergistic effects of multiple pathways.

### 3.5. Molecular Docking Results

**Figure 8:**
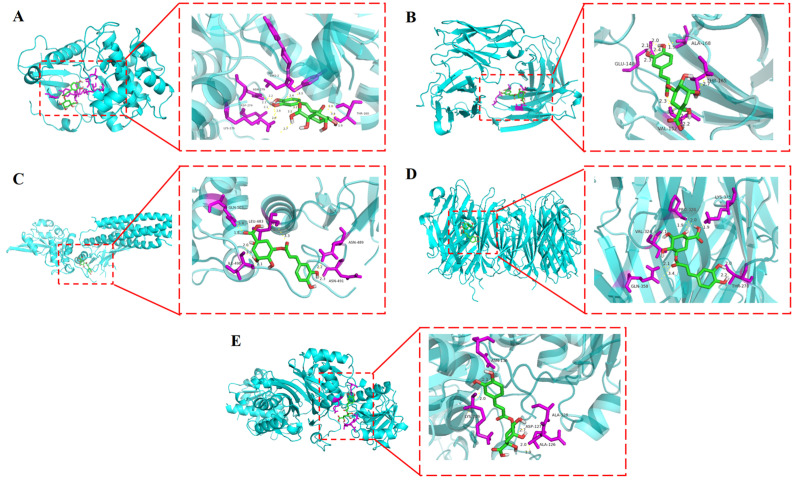
Visualization results of molecular docking between CGA and AKT1 (**A**), EGFR (**B**), STAT3 (**C**), TNF (**D**), and GAPDH (**E**). [H-bond (yellow dotted line)].

Docking simulation and visualization analysis were performed between chlorogenic acid and the five targets (GAPDH, AKT1, TNF, EGFR, and STAT3) screened in Section 3.4.2. The results showed that chlorogenic acid had binding energies of less than −6.3 kcal·mol^−1^ with GAPDH, TNF, and AKT1, indicating good binding interactions. CGA could act on multiple core targets simultaneously (Figure 8). It bound to the active pockets of these targets through mechanisms such as hydrogen bonds and hydrophobic interactions, suggesting that its hypoglycemic effect might be achieved through multi-target synergy. Specifically, CGA forms a stable complex with AKT1, where its hydroxyl groups interact with residues such as ASN-279, ASP-274, LYS-276, and THR-160. This study provides a theoretical basis for developing value-added products from sweet potato leaves based on the hypoglycemic properties of CGA. However, further experimental studies are essential to verify its translational potential.

### 3.6. Effect of Crude Extract of Chlorogenic Acid from Sweet Potato on Survival Rate and Glucose Production of Primary Mouse Hepatocytes

#### 3.6.1. Effect of CGA Crude Extract from Sweet Potato Leaves on Survival and Gluconeogenesis of Primary Mouse Hepatocytes

**Figure 9:**
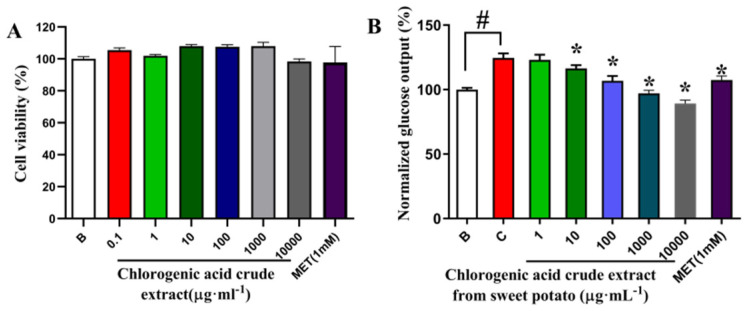
Crude extract of chlorogenic acid from sweet potato inhibited hepatic glucose output in primary mouse hepatocytes. (**A**) MTT cell proliferation to assess the number of viable cells; (**B**) hepatic glucose output results. Data were expressed as the mean ± SD (n = 6). * *p* < 0.05 vs. glucagon, # *p* < 0.05 vs. blank. CGA, chlorogenic acid; MET, metformin.

The effect of the CGA crude extract on primary hepatocyte viability was assessed using the MTT assay. As shown in Figure 9A, the extract, at concentrations ranging from 0.1 to 10,000 µg·mL^−1^, showed no significant impact of sweet potato CGA crude extract on cell proliferation. This confirmed the non-cytotoxic nature of the extract within this concentration range, allowing its use in subsequent experiments. At the cellular level, we further explored the inhibitory effect of CGA crude extract on the hepatic glucose output of primary cells, using the hepatic glucose output rate of primary mouse hepatocytes in the blank group as 100%. As shown in Figure 9B, CGA crude extract exhibited a significant inhibitory effect at a concentration of 10 µg·mL^−1^, and this effect increased in a concentration-dependent manner. These results suggest that CGA crude extract can reduce hepatic gluconeogenesis induced by glucagon.

#### 3.6.2. Effects of Sweet Potato CGA Crude Extract on Pyruvate Tolerance in Mice

**Figure 10:**
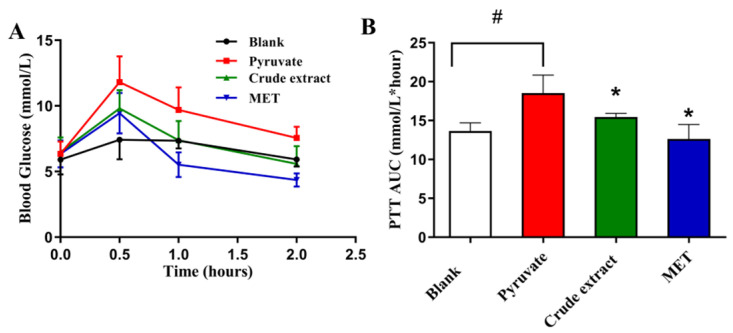
Crude extract of CGA from sweet potato leaves inhibits endogenous glucose production in acute animal mice. (**A**) Blood glucose change value in mice; (**B**) pyruvate tolerance in mice. Data were expressed as the mean ± SD (n = 6). * *p* < 0.05 vs. pyruvate, # *p* < 0.05 vs. blank. CGA, chlorogenic acid (150 mg/kg); MET, metformin (200 mg/kg).

Pyruvate is a substrate for gluconeogenesis in the body, which raises blood glucose. As fasting blood glucose is mainly regulated by endogenous gluconeogenesis, the pyruvate tolerance test serves as an indicator of hepatic glucose production. Blood glucose changes were monitored for 2 h, and the area under the curve (AUC) was calculated (Figure 10A,B). In all groups, the blood glucose level of mice reached its maximum at 0.5 h and returned to baseline approximately 2 h after pyruvate administration. The model group was always higher than that of the other three groups, and the blood glucose value could fall back to the normal level after the administration of chlorogenic acid; i.e., the crude extract of CGA could significantly reduce glucose produced by hepatic gluconeogenesis caused by sodium pyruvate.

## 4. Discussion

The methanol extract of sweet potato leaves contains the highest phenolic acid content, followed by the peel, whole root, and fleshy tissue [35]. Based on a previous report indicating that September offers the highest chlorogenic acid (CGA) content [36], sweet potato leaves harvested in this month were selected for the study. Common methods for extracting CGA include water decoction [37], complete enzymatic hydrolysis [38], supercritical carbon dioxide extraction [39], and ultrasonic-assisted extraction (UAE) [40]. The decoction extraction method is characterized by low cost, minimal equipment requirements, and simplicity, but it has a long duration, produces many impurities, and the effectiveness of extraction is influenced by the inherent properties of the plant. The cost of the supercritical carbon dioxide extraction and complete enzymatic hydrolysis is too high, and the enzyme activity is easily affected. UAE is simple, fast, time-efficient, and has a high yield, so this method was selected for extracting CGA from sweet potato leaves.

Studies have shown that the polyphenols, anthocyanins, and flavonoids in sweet potato leaves have blood sugar-lowering effects [41,42]. CGA is an active ingredient in many medicinal herbs [43] and has hypoglycemic effects. Although chlorogenic acid does not directly affect the digestion of carbohydrates, it may affect the absorption and subsequent utilization of glucose [44] through the effect of metabolites produced by endogenous pathways or intestinal flora, and chlorogenic acid [45] increases the expression of CPT1a, ACOX1, ATGL, and HSL and decreases the expression of MGAT1, DGAT1, DGAT2, CD36, and FATP4 to reduce liver lipid content. In addition, CGA can reduce body weight, improve glucose tolerance and insulin resistance, and has a prominent hypoglycemic effect [46,47]; thus, a network pharmacology approach proved to be a feasible and valuable strategy for systematically analyzing the potential active ingredients, key targets, and signaling pathways of sweet potato leaves in lowering blood glucose. This study, based on KEGG pathway enrichment analysis, further confirmed that the main pathways through which diabetes is intervened involve lipid and atherosclerosis, the PI3K-Akt signaling pathway, and the TNF signaling pathway, which is consistent with previous mechanistic understandings [48]. It was found that nine active ingredients in sweet potato leaves act on 90 targets related to diabetes, with the main targets for their blood glucose-lowering effects being AKT1, GAPDH, STAT3, TNF, and EGFR. AKT is a key mediator of insulin signaling, promoting cellular uptake and utilization of glucose. Accordingly, studies have demonstrated that supplementation of an HFD with CGA improves glucose and lipid metabolism disorders by regulating the AMPK/Akt pathway [49]. TNF-*α* is associated with diabetic vascular complications [50], and Arctii Fructus lignans alleviate diabetic complications by reducing oxidative stress, regulating TGF-*β*/VEGF signaling, and restoring autophagy balance [51].

The existing literature on the hypoglycemic mechanisms of CGA has predominantly focused on insulin signaling pathways [52], with comparatively limited investigation into its modulatory effects on glucagon-mediated regulation. Consequently, this study is designed to elucidate the protective role and underlying molecular mechanisms of chlorogenic acid in counteracting glucagon-induced hepatic glucose production. The results showed that CGA could significantly inhibit the production of glucose in primary mouse hepatocytes at 10.00 μg·mL^−1^. In acute animal tests, mice administered CGA or a crude extract of CGA improved the increase in blood glucose caused by glucagon or sodium pyruvate, and in agreement with the study of Abdollahi Milad et al., CGA supplementation can improve prediabetic complications [28], including weight gain and elevated fasting blood glucose and plasma insulin levels, and these effects are related to changes in mRNA levels of important genes such as hepatic glycogen synthesis, glycogenesis, and glycolysis, suggesting that chlorogenic acid has important significance in inhibiting diabetes. Despite China’s long history of using plants rich in CGA and its derivatives, key challenges persist. These include not only the insufficient mechanistic exploration but also the lack of effective strategies to maximize plant utilization and minimize waste. Addressing these issues is crucial for future research and application.

## 5. Conclusions

In this study, the optimal process conditions for extracting CGA from sweet potato leaves was determined using a single-factor approach combined with a Box–Behnken response surface methodology, with ethanol as the solvent. Our findings indicate that CGA from sweet potato leaves exerts hypoglycemic effects through multiple targets and pathways. CGA crude extract was non-toxic to primary mouse hepatocytes within the tested concentration and significantly suppressed glucagon-induced glucose production in hepatocytes. This inhibitory effect was confirmed in an acute mouse model, where the extract effectively reduced the glucose production. This study contributes to reducing the waste of sweet potato leaf resources and provides a theoretical basis for the development of sweet potato leaf resources as hypoglycemic health products or medicines.

## Figures and Tables

**Table 1:** Single-factor experimental factors and levels.

Design	Ultrasonic Time (A)/min	Solid–Liquid Ratio (B)/(g/mL)	Ethanol Concentration (C)/%
1	10, 20, 30, 40, 50	1:30	70
2	30	1:10, 1:20, 1:30, 1:40, 1:50	70
3	30	1:30	50, 60, 70, 80, 90

**Table 2:** Response surface factor levels.

Level	A/(min)	B/(g/mL)	C/(%)
−1	10	1:10	50
0	30	1:30	70
1	50	1:50	90

**Table 3:** Response surface test design results.

Number	A	B	C	Yield of CGA (Y)/%
1	−1	−1	0	3.0903 ± 0.0932
2	1	−1	0	2.5221 ± 0.2194
3	−1	1	0	2.3798 ± 0.0755
4	1	1	0	3.5263 ± 0.0518
5	−1	0	−1	2.7959 ± 0.1027
6	1	0	−1	2.6667 ± 0.1093
7	−1	0	1	2.3326 ± 0.0551
8	1	0	1	2.4675 ± 0.0787
9	0	−1	−1	3.0138 ± 0.7027
10	0	1	−1	3.3620 ± 0.0574
11	0	−1	1	2.1054 ± 0.1347
12	0	1	1	2.5717 ± 0.0316
13	0	0	0	3.5726 ± 0.0259
14	0	0	0	3.7070 ± 0.0698
15	0	0	0	3.7894 ± 0.0385
16	0	0	0	3.8841 ± 0.0356
17	0	0	0	3.6392 ± 0.0189

**Table 4:** Analysis of the extraction yield of CGA crude extracts.

Source	Sum of Squares	Mean Square	df	*F*-Value	*p*-Value	Significant
Model	5.2560	9	0.5840	15.1831	0.0008	**
A	0.0426	1	0.04263	1.1083	0.3274	
B	0.1536	1	0.1536	3.9921	0.0859	
C	0.6969	1	0.6969	18.1177	0.0038	**
AB	0.7350	1	0.7350	19.1090	0.0033	**
AC	0.0174	1	0.0174	0.4535	0.5223	
BC	0.0035	1	0.0035	0.0907	0.7720	
A^2^	1.1305	1	1.1305	29.3923	0.0010	**
B^2^	0.4329	1	0.4329	11.2550	0.0122	*
C^2^	1.6957	1	1.6957	44.0849	0.0003	**
Residual	0.2692	7	0.0385			
Lack of fit	0.2091	3	0.0697	4.6358	0.0863	
Pure error	0.0601	4	0.0150			
Cor total	5.5252	16				

Notes: “*” indicates significant (*p* < 0.05) and “**” indicates extremely significant (*p* < 0.01). R^2^ = 0.954039932, Radj^2^ = 0.8865.

**Table 5:** Validation of response surface results.

Number	Ultrasonic Time (min)	Solid–Liquid Ratio (g/mL)	Ethanol Concentration (%)	Yield of CGA (%)
1	50	1:40	65	3.53
2	50	1:40	65	3.56
3	50	1:40	65	3.51

**Table 6:** Main active components of sweet potatoes leaves.

No	Component	MV	OB (%)	DL
MOL000414	Caffeic acid	180.16	54.97	0.05
MOL000098	Quercetine	302.24	46.43	0.28
MOL000422	Kaempferol	286.24	41.88	0.24
MOL005889	Rhamnetin	316.26	36.36	0.32
MOL013377	Lutein	568.87	33.92	0.58
MOL000561	Astragalin	448.38	14.03	0.74
MOL001955	Chlorogenic acid	354.31	11.93	0.33
MOL012788	Tiliroside	594.52	1.94	0.66
MOL000437	Isoquercitrin	464.38	1.87	0.77

## Data Availability

No new data were created or analyzed in this study.
